# Global circular RNA expression profile of human gastric cancer and its clinical significance

**DOI:** 10.1002/cam4.1055

**Published:** 2017-05-23

**Authors:** Yongfu Shao, Jinyun Li, Rongdan Lu, Tianwen Li, Yunben Yang, Bingxiu Xiao, Junming Guo

**Affiliations:** ^1^ Department of Biochemistry and Molecular Biology and Zhejiang Key Laboratory of PathophysiologyMedical School of Ningbo UniversityNingbo315211China; ^2^ Department of Gastroenterologythe Affiliated Hospital of Medical School of Ningbo UniversityNingbo315020China

**Keywords:** Biomarker, circular RNA, clinical significance, expression profile, gastric cancer

## Abstract

Circular RNAs (circRNAs) are a new class of noncoding RNAs. However, the expression profile and clinical significance of circRNAs in human gastric cancer is unclear. The global circRNA expression profile in human gastric cancer was measured by circRNA microarray. Hsa_circ_0014717, one of the most downregulated circRNAs in microarray, was selected as a targeted circRNA to explore its levels in gastric tissues and gastric juice. Freeze‐thaw experiment and incubation experiment confirmed the stability of gastric juice circRNAs. A total of 308 circRNAs, including 107 (34.74%) upregulated and 201 (65.26%) downregulated circRNAs, were found significantly aberrantly expressed in gastric cancer tissues. The top ten upregulated in gastric cancer tissues were hsa_circ_0035445, hsa_circ_0003789, hsa_circ_0063809, hsa_circ_0074362, hsa_circ_0006282, hsa_circ_0011107, hsa_circ_0084606, hsa_circ_0005556, hsa_circ_0050547, and hsa_circ_0006470, while the top ten downregulated ones were hsa_circ_0007099, hsa_circ_0001897, hsa_circ_0007707, hsa_circ_0008832, hsa_circ_0001546, hsa_circ_0002089, hsa_circ_0004680, hsa_circ_0000154, hsa_circ_0004458, and hsa_circ_0008394. The hot‐point chromosomes were chr1, chr2, chr3, chr9, and chr17. Hsa_circ_0014717 was significantly downregulated in 77.2% (74/96) gastric cancer tissues. Its levels in gastric cancer tissues were related to tumor stage (*P *= 0.037), distal metastasis (*P *= 0.048), tissue carcinoembryonic antigen (*P *= 0.001), and carbohydrate antigen 19‐9 expression (*P *= 0.021). More importantly, hsa_circ_0014717 can stably exist in human gastric juice; and its nature meets the requirements of clinical detection. Our study uncovered the circRNA expression profile in human gastric cancer. Moreover, some circRNAs can stably exist in human body fluid, and has the potential to be used as novel biomarkers for the screening of high‐risk gastric cancer patients.

## Introduction

Gastric cancer is the fifth most common malignancy and the third leading cause of global cancer death of people in the world 1. Despite recent advances in surgical techniques and combined chemotherapy strategies, the total treatment effectiveness of advanced gastric cancer have not shown any significant improvement and the 5‐year overall survival rate is still very low 2, 3. These results are mainly due to molecular diversity that causes high clinical heterogeneity even in similar clinical and pathologic features 4. Thus, for a better understanding of gastric cancer heterogeneity and to develop personalized therapeutic strategies, reliable identification of detailed molecular characterization of gastric cancer tissues is critical.

Circular RNAs (circRNAs) are special class of endogenous noncoding RNAs that are formed by back‐splicing events via exon or intron circularization 5. Their features are conservation, stability, abundance, and tissue‐specific expression in organisms 6, 7, 8. Many studies have reported that circRNAs have numerous biologic functions, which are widely involved in forming RNA‐protein complexes, acting as microRNA sponges, and regulating targeted gene transcription and splicing 7, 9, 10. Several associations between altered circRNAs and human diseases have also been discovered 11, 12, 13. However, the roles of circRNAs in cancer pathological processes, especially in gastric carcinogenesis, are largely unknown.

Since the global circRNAs expression profile in human gastric cancer has not been uncovered, in this study, we used circRNA microarray to investigate the differential expression profiles of circRNAs between gastric cancer tissues and paired noncancerous tissues. We then selected hsa_circ_0014717, one of the middle downregulated circRNAs in microarray screening, as a targeted circRNA to explore its clinical significance and application in gastric cancer. Its gene is located at chr1:156290629‐156304709 with a spliced length of 516 nt. Our results showed that some circRNAs, such as hsa_circ_0014717, can stably exist in human body fluid, and has the potential application in the screening of gastric cancer.

## Materials and Methods

### Patients and specimens

Patients were collected from the center for gastroenterology of the Affiliated Hospital of Medical School of Ningbo University (China) between February 2011 and February 2016. Gastric cancer tissues and their matched adjacent nontumorous tissues were obtained from 96 surgical patients. Gastric juice samples were collected from 38 healthy volunteers, 30 gastric ulcer patients, 15 chronic atrophic gastritis patients, and 39 gastric cancer patients during endoscopic examination. No patient received medical treatment before endoscopy examination or surgery. The final diagnosis of each patient was confirmed histopathologically. All specimens collection and preprocessed were according to previously described protocol and preserved at −80°C condition until RNA isolation 14.

Tumors were classified following the tumor‐node‐metastasis (TNM) staging system (7th ed.). Histologic grade was assessed following the National Comprehensive Cancer Network (NCCN) Clinical Practice Guideline of Oncology (V.1.2012). This study was approved by the Human Research Ethics Committee of Ningbo University; and informed consent was obtained from all participants. Double‐blind manner was used through the entire process of all clinical samples and data collection.

### Total RNA extraction and reverse transcription reaction

Tissue total RNA was extracted using TRIzol reagent (Ambion, Carlsbad, CA), whereas gastric juice samples were processed using TRIzol LS reagent (Ambion). All steps of RNA extraction were followed as per the manufacturer's instructions. The concentrations of total RNA were then determined using a DS‐11+ Spectrophotometer (DeNovix, Wilmington, DE). The integrity of RNA was assessed by 1% agarose gel electrophoresis. Finally, total RNA was reverse transcribed to cDNA by GoScript Reverse Transcription (RT) System (Promega, Madison, WI) following the manufacturer's protocol.

### CircRNA microarray analysis

Three gastric cancer tissues and their matched adjacent nontumorous tissues 5 cm away from the edge of tumor were selected to analyze circRNA expression profile using Arraystar Human circRNA Array (Arraystar, Rockville, MD). Total RNA from six samples were amplified and transcribed into fluorescent cRNA utilizing random primer according to Arraystar's Super RNA Labeling protocol (Arraystar). The labeled cRNAs were hybridized onto the Arraystar Human circRNA Array (6 × 7K, Arraystar), and incubated for 17 h at 65°C in an Agilent Hybridization Oven (Agilent Technologies, Santa Clara, CA). After having washed the slides, the arrays were scanned by the Axon GenePix 4000B microarray scanner (Molecular Devices, Sunnyvale, CA).

Scanned images were then imported into GenePix Pro 6.0 software (Axon) for grid alignment and data extraction. Quantile normalization and subsequent data processing were performed using the R software package. Differentially expressed circRNAs with statistical significance between two groups were identified through Fold Change filtering or Volcano Plot filtering. Hierarchical clustering was performed to show the distinguishable circRNA expression pattern among samples.

### Quantitative detection of targeted circRNA

The real‐time quantitative reverse transcription‐polymerase chain reaction (qRT‐PCR) was performed using GoTaq qPCR Master Mix (Promega) on an Mx3005P Real‐Time PCR System (Stratagene, La Jolla, CA following the manufacturer's instructions. Divergent primers of targeted circRNA hsa_circ_0014717 and convergent primers of glyceraldehyde 3‐phosphate dehydrogenase (GAPDH) were designed and synthesized by Sangon Biotech (Shanghai, China), respectively. The use of divergent primers can only amplify circRNA and differentiates the contamination from its linear isoforms**.** The sequences of hsa_circ_0014717 and GAPDH were as follows: 5′‐TTGCCCTGGATGCTGTCAAG‐3′ and 5′‐GGTCATCACAATGCCTCCCAT‐3′ for hsa_circ_0014717; 5′‐ACCCACTCCTCCACCTTTGAC‐3′ and 5′‐TGTTGCTGTAGCCAAATTCGTT‐3′ for GAPDH. Targeted circRNA expression levels were calculated using the Δ*C*_t_ method with GAPDH as the control 13. Lower Δ*C*_t_ values indicate higher expression levels.

### Cloning and sequencing of qRT‐PCR products

qRT‐PCR products of hsa_circ_0014717 in gastric juice were purified using the UNIQ‐10 PCR Product Purification Kit (Sangon), and then cloned into the pUCm‐T vector (Sangon) following the manufacturer's instructions. DNA sequencing was performed by Sangon Biotech Company, Ltd.

### Statistical analysis

Statistical analyses were performed using Statistical Program for Social Sciences (SPSS) 20.0 software (SPSS, Chicago, IL). Student's *t*‐test and one way analysis of variance (ANOVA) test were flexibly used according to actual conditions. *P *< 0.05 was considered as statistically significant.

## Results

### Circular RNA expression profile in gastric cancer

**Figure 1:**
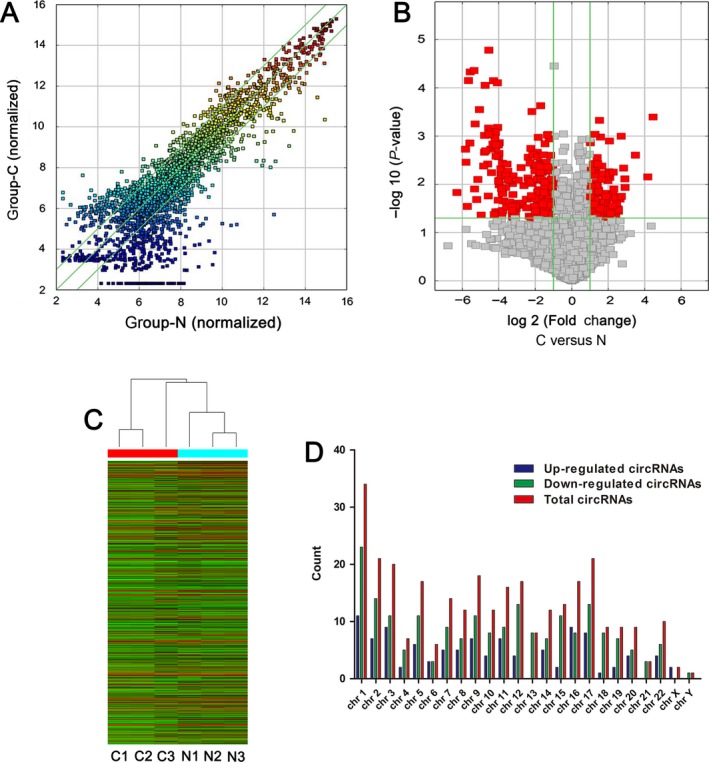
Differences and characterizations of circRNA expression profile between gastric cancer tissues and paired adjacent nontumorous tissues. (A) Scatter plots are used to evaluate the difference of circRNA expression between gastric cancer tissues and paired adjacent nontumorous tissues. (B) Volcano plots are used to visualize the differential circRNA expression between gastric cancer tissues and paired adjacent nontumorous tissues. The red points in plot represent the differentially expressed circRNAs with statistical significance. (C) Hierarchical cluster analysis of expressed circRNAs in three gastric cancer tissues and paired adjacent nontumorous tissues. (D) Chromosomal distributions of differentially expressed circRNAs in gastric cancer tissues and paired adjacent nontumorous tissues.

High‐throughput human circRNA microarray was used to assess the differences of circRNA expression profiles between gastric cancer tissues and paired adjacent nontumorous tissues. A total of 5396 circRNAs were detected (data accessible at NCBI GEO database, accession GSE89143, Guo, 2016; https://www.ncbi.nlm.nih.gov/geo/query/acc.cgi?acc=GSE89143). Differentially expressed circRNAs with statistical significance between cancer group and nontumorous group were identified through Fold Change filtering (Fig. 1A) or Volcano Plot filtering (Fig. 1B). Hierarchical clustering was performed to show the distinguishable circRNA expression pattern among samples (Fig. 1C). CircRNA shaping fold changes ≥2.0 and *P* ≤ 0.05 were selected as the significantly differentially expressed ones. Our microarray data showed that the expression of circRNAs in gastric cancer tissues was significantly different from that in matched nontumor tissues. A total of 308 significantly differential expressed circRNAs were found. Among them, 107 and 201 circRNAs were upregulated and downregulated, respectively, (GEO No. GSE89143). Downregulated circRNAs (65.26%) were more common than upregulated circRNAs (34.74%) in microarray data. The top 10 upregulated (hsa_circ_0035445, hsa_circ_0003789, hsa_circ_0063809, hsa_circ_0074362, hsa_circ_0006282, hsa_circ_0011107, hsa_circ_0084606, hsa_circ_0005556, hsa_circ_0050547, and hsa_circ_0006470) and downregulated (hsa_circ_0007099, hsa_circ_0001897, hsa_circ_0007707, hsa_circ_0008832, hsa_circ_0001546, hsa_circ_0002089, hsa_circ_0004680, hsa_circ_0000154, hsa_circ_0004458, and hsa_circ_0008394) circRNAs in gastric cancer tissues are listed in Table 1. Moreover, the distributions of differentially expressed circRNAs in human chromosomes showed that most circRNAs were transcribed from chr1, chr2, chr3, chr9, and chr17, but seldom from chr21, chrX, and chrY (Fig. 1D).

**Table 1:** The top 10 upregulated and downregulated circRNAs in gastric cancer tissues comparing with paired nontumorous tissues

CircRNA ID	Chromosome	Regulation	Fold change	Strand	Gene symbol	*P*‐value
hsa_circ_0035445	chr15	Up	21.95	‐	*ALDH1A2*	0.00040
hsa_circ_0003789	chr2	Up	17.88	+	*TSN*	0.00698
hsa_circ_0063809	chr22	Up	11.20	‐	*CELSR1*	0.00249
hsa_circ_0074362	chr5	Up	7.58	+	*ARHGAP26*	0.01156
hsa_circ_0006282	chr8	Up	7.50	‐	*TCEB1*	0.00770
hsa_circ_0011107	chr1	Up	7.16	‐	*RPA2*	0.00459
hsa_circ_0084606	chr8	Up	6.56	‐	*ASPH*	0.01775
hsa_circ_0005556	chr2	Up	6.54	‐	*NBAS*	0.00100
hsa_circ_0050547	chr19	Up	6.25	+	*UBA2*	0.02015
hsa_circ_0006470	chr1	Up	5.90	+	*MFN2*	0.03360
hsa_circ_0007099	chr15	Down	78.86	+	*ABHD2*	0.01473
hsa_circ_0001897	chr9	Down	56.72	+	*POMT1*	0.00186
hsa_circ_0007707	chr10	Down	55.67	+	*LCOR*	0.00347
hsa_circ_0008832	chr22	Down	52.90	+	*FBXO7*	0.02867
hsa_circ_0001546	chr5	Down	50.73	‐	*FAM114A2*	0.00007
hsa_circ_0002089	chr11	Down	48.27	+	*ARHGEF12*	0.00139
hsa_circ_0004680	chr1	Down	47.54	‐	*CCT3*	0.00005
hsa_circ_0000154	chr1	Down	41.07	+	*DCAF6*	0.00004
hsa_circ_0004458	chr8	Down	40.26	‐	*PSD3*	0.01244
hsa_circ_0008394	chr3	Down	39.40	+	*TIMMDC1*	0.00056

### Expression of hsa_circ_0014717 was downregulated in gastric cancer tissues

**Figure 2:**
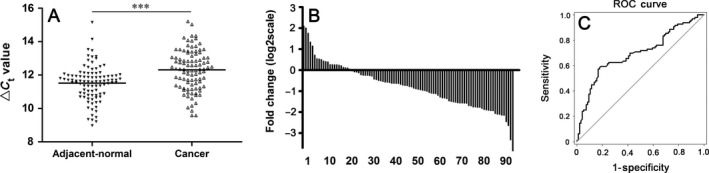
Hsa_circ_0014717 expression levels in gastric cancer tissues. (A) Hsa_circ_0014717 levels in 96 gastric cancer tissues and their matched adjacent nontumorous tissues were detected uisng qRT‐PCR method. Larger Δ*C*_t_ value indicates lower expression. ***P < 0.001. (B) Hsa_circ_0014717 levels were significantly downregulated in 77.2% (74/96) of gastric cancer tissues. (C) ROC curve was built for differentiating gastric cancer tissues from controls. The area under the curve was up to 0.696. ROC, Receiver operating characteristic.

Next, to validate the microarray results, we expanded the sample size. Considering that more than two thirds of significantly aberrantly expressed circRNAs in gastric cancer tissues were downregulated ones, and that the most upregulated and downregulated of circRNA in gastric cancer tissues was 21.95 folds and 78.86 folds (Table 1), we chose hsa_circ_0014717, which was one of the middle downregulated circRNAs in gastric cancer and its fold change (23.4‐fold) was similar to that (21.95‐fold) of the most upregulated circRNA, to validate the accuracy of microarray results. Its expression levels in 96 gastric cancer tissues and their matched adjacent nontumorous tissues were measured by qRT‐PCR method (Fig. 2A). The results showed that hsa_circ_0014717 expression was significantly downregulated in 77.2% (74/96) gastric cancer tissues compared with the adjacent nontumorous tissues (*P *< 0.001, Fig. 2B). Receiver operating characteristic (ROC) curve was built to differentiate gastric cancer tissues from controls. The area under the curve (AUC) was up to 0.696 (95% CI: 0.621–0.771; *P *< 0.001; Fig. 2C). The optimal cutoff value of hsa_circ_0014717 was 12.14, with sensitivity and specificity were 59.38% and 81.25%, respectively. The false positive rate and false negative rate were 18.75% and 40.62%, respectively. The positive predictive value and negative predictive value were 76.00% and 67.24%, respectively. Our data indicated that the expression of hsa_circ_0014717 in lager size of sample number was consistent with that of microarray analysis.

Then, analyses were performed to assess the relationship between hsa_circ_0014717 expression levels and gastric cancer patients' clinical features. As shown in Table 2, its expression levels in gastric cancer tissues were significantly related to tumor stage (*P *= 0.037), distal metastasis (*P *= 0.048), tissue carcinoembryonic antigen (CEA) (*P *= 0.001), and carbohydrate antigen 19‐9 (CA19‐9) expression (*P *= 0.021). However, they were not associated with other clinic pathologic factors such as tumor diameter, lymphatic metastasis, invasion, and cell differentiation.

**Table 2:** Relationship of Hsa_circ_0014717 expression levels (Δ*C*_t_) in cancer tissues with clinicopathological factors of gastric cancer patients

Characteristics	No. of case (%)	Mean ± SD	*P* value
Age (year)			
≥60	61 (63.5)	12.343 ± 1.253	0.727
<60	35 (36.5)	12.254 ± 1.109	
Gender			
Male	65 (67.7)	12.239 ± 1.274	0.398
Female	31 (32.3)	12.461 ± 1.002	
Tumor location			
Sinuses ventriculi	49 (51.1)	12.342 ± 1.064	0.629
Cardia	10 (10.4)	12.110 ± 1.469	
Corpora ventriculi	25 (26.0)	12.158 ± 0.928	
Others	12 (12.5)	12.664 ± 1.889	
Diameter (cm)			
≥5	47 (49.0)	12.358 ± 1.332	0.706
<5	49 (51.0)	12.265 ± 1.065	
Differentiation			
Well	12 (12.5)	12.765 ± 1.184	0.240
Moderate	47 (49.0)	12.355 ± 1.258	
Poor	37 (38.5)	12.106 ± 1.104	
Stage			
Early	24 (25.0)	12.750 ± 1.332	**0.037**
Advanced	72 (75.0)	12.164 ± 1.190	
Borrmann type			
I & II	19 (26.4)	11.976 ± 1.234	0.426
III & IV	53 (73.6)	12.231 ± 1.178	
Pathologic diagnosis			
Signet ring cell cancer	15 (15.6)	12.143 ± 0.995	0.559
Adenocarcinoma	81 (84.4)	12.341 ± 1.234	
Invasion			
T_1_ & T_2_	36 (37.5)	12.554 ± 1.154	0.123
T_3_ & T_4_	60 (62.5)	12.164 ± 1.209	
Lymphatic metastasis			
N _0_	38 (39.6)	12.266 ± 1.314	0.768
N _1‐3_	58 (60.4)	12.340 ± 1.126	
Distal metastasis			
M_0_	82 (85.4)	12.406 ± 1.179	**0.048**
M_1_	14 (14.6)	11.750 ± 1.191	
Venous invasion			
Absent	53 (55.2)	12.207 ± 1.386	0.329
Present	43 (44.8)	12.438 ± 0.914	
Perineural invasion			
Absent	47 (49.0)	12.543 ± 1.193	**0.062**
Present	49 (51.0)	12.087 ± 1.171	
Carcinoembryonic antigen(tissue)			
Positive	74 (77.1)	12.586 ± 1.081	**<0.001**
Negative	22 (22.9)	11.385 ± 1.123	
CA19‐9 (Tissue)			
Positive	54 (56.3)	12.557 ± 1.161	**0.021**
Negative	42 (43.7)	11.993 ± 1.183	

Bold values: *P* < 0.05.

### Hsa_circ_0014717 stably exists in human gastric juice and significantly decreased in chronic atrophic gastritis group

**Figure 3:**
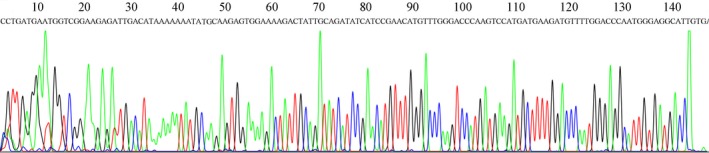
Representative sequencing results of the products of qRT‐PCR from the detection of hsa_circ_0014717 in gastric juice.

**Figure 4:**
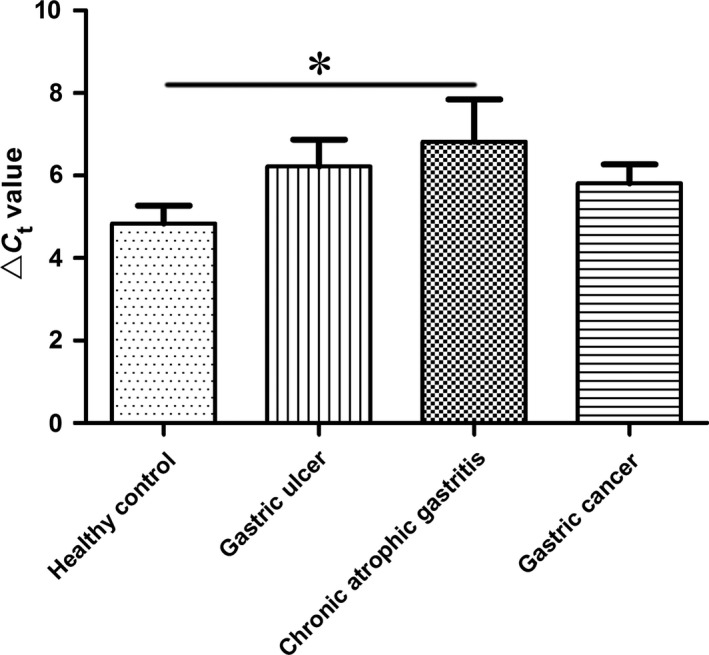
Hsa_circ_0014717 levels in gastric juices. Hsa_circ_0014717 levels in gastric juices from various stages of gastric carcinogenesis including healthy controls (*n* = 38), patients with gastric ulcers (*n* = 30), chronic atrophic gastritis (*n* = 15) and gastric cancer (*n* = 39) were detected by qRT‐PCR. Larger Δ*C*_t_ value indicates lower expression.*P < 0.05.

**Figure 5:**
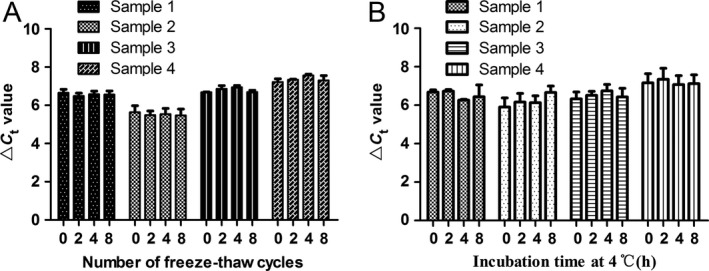
Freeze‐thaw experiments and incubation experiments confirmed the stability of hsa_circ_0014717 in gastric juice. (A) Freeze‐thaw experiments. Four gastric juice samples were randomly selected and then equally divided to four parts. After 0, 2, 4, and 8 cycles of freeze‐thaw, the hsa_circ_0014717 levels were detected by qRT‐PCR. Three independent experiments were performed. *P *> 0.05. (B) Incubation experiments. Four gastric juice samples were first randomly selected and then equally divided to four parts. Then, they were stored at 4°C. After 0, 2, 4, and 8 h incubation, the hsa_circ_0014717 levels were detected by qRT‐PCR. Three independent experiments were performed. *P *> 0.05.

Gastric juice has significant advantage in reflecting gastric cancer for its high specificity of gastric organ; and the use of gastric juice in disease diagnosis is a non‐invasion method 15, 16. In this study, we further explored whether gastric juice hsa_circ_0014717 could be used as a biomarker for screening gastric cancer patients. First, the sequencing results of the qRT‐PCR products of gastric juice confirmed the existence of hsa_circ_0014717 in gastric juice (Fig. 3). Then, from the results of 38 healthy persons, 30 gastric ulcer patients, 15 chronic atrophic gastritis patients and 39 gastric cancer patients, we unexpectedly found that there was no significant difference of gastric juice hsa_circ_0014717 levels among healthy group, gastric ulcer group, and gastric cancer group, but there was a sharp decline in chronic atrophic gastritis group (Fig. 4). Moreover, our data also showed that there were no significant differences of hsa_circ_0014717 levels among gastric juice of up to eight cycles of freeze‐thaw or under different time points (0, 2, 4, and 8 h) at 4°C (Fig. 5).

## Discussion

CircRNAs are a special class of endogenous RNAs. Recent studies have indicated that dysregulated circRNAs are associated with several human diseases, such as nervous system, cardiovascular system diseases and cancers 8, 9, 11, 12, 17. Some altered circRNAs are demonstrated to associate with tumor development, invasion, metastasis or patient prognosis 17, 18, 19. Shang et al. found that hsa_circ_0005075 exhibited significant difference in global circRNA expression profile between hepatocellular carcinoma and healthy control 17. Its expression correlates with tumor size and participates in cell adhesion during cancer development 17. Song et al. 18 reported that 476 circRNAs were differentially expressed in control brain tissues and gliomas. Su et al. 20 discovered 57 and 17 circRNAs significantly upregulated and downregulated in human radioresistant esophageal cancer, respectively. However, the global expression profile and its clinical significance of circRNAs in human gastric cancer is not well known.

In this study, we first investigated circRNA expression profile in human gastric cancer by high‐throughput circRNA microarray. Our results showed that circRNA expression in gastric cancer is significantly different from that in healthy control (Fig. 1). Compared with nontumor tissues, our microarray data showed a total of 308 circRNAs were significantly dysregulated in gastric cancer tissues. By searching the literatures from PubMed (https://www.ncbi.nlm.nih.gov/pubmed/), before November 26, 2016, all the top 10 upregulated and top 10 downregulated circRNAs were not found aberrantly expressed in cancer tissues. Moreover, the distributions of differentially expressed circRNAs in human chromosomes showed that most circRNAs are transcribed from chr1, chr2, chr3, chr9, and chr17 (Fig. 1D). All these findings strongly suggest distinct gene expression patterns of circRNA in gastric cancer and its important role in pathophysiology.

Some clinicopathological features such as tumor stage, and distal metastasis, tissue CEA and CA19‐9 expression appear as independent prognostic factors affecting gastric cancer‐free survival and overall survival 21, 22, 23. For example, the 5‐year survival rates for gastric cancer patients with pTNM stage I, II, III, and IV were 93.2%, 72.4%, 39.1%, and 5.2%, respectively 21. Tissue CEA expression is significantly correlated with gastric cancer invasion, metastasis, and TNM staging 22. Patients with negative tissue CEA staining often have a better prognosis. The 5‐year survival rates of gastric cancer patients were 67.6%, 53.9%, and 40.1% for negatively, moderately, and intensely positively stained tissues CEA, respectively 22. In our study, hsa_circ_0014717 was selected as a targeted circRNA to validate the accuracy of microarray results. Our results showed that hsa_circ_0014717 was significantly downregulated in 77.2% (74/96) gastric cancer tissues; and the AUC of hsa_circ_0014717 was up to 0.696 (Fig. 2). More importantly, considering clinicopathological factors, we found that hsa_circ_0014717 expression levels in gastric cancer tissues were related to tumor stage, distal metastasis, tissue CEA and CA19‐9 expression (Table 2), which are independent prognostic factors in gastric cancer patients. This indicated that circRNAs have the potential for clinical prognosis prediction.

Body fluids are the commonly used materials in the diagnosis of human diseases. Gastric juice has significant advantage in reflecting gastric diseases 14, 16, 24. In this study, we further explored whether circRNAs could be used as biomarkers for gastric diseases screening, particularly for early gastric cancer. We first confirmed the existence of hsa_circ_0014717 in gastric juice (Fig. 3). Chronic atrophic gastritis is considered as an independent risk factor for gastric cancer 25. People with chronic atrophic gastritis have been thought as high‐risk of gastric cancer. In this study, we found that there was a significant difference between healthy group and chronic atrophic gastritis group (Fig. 4). The hsa_circ_0014717 levels in gastric juice from patients with chronic atrophic gastritis were obviously decreased. More importantly, results of freeze‐thaw and incubation experiments confirmed the stability of gastric juice hsa_circ_0014717 (Fig. 5). These implies that some circRNAs such as hsa_circ_0014717 can exist in human body fluid and has the potential to be used as biomarker for disease screening.

In conclusion, our study uncovered the changes of circRNAs expression profile in human gastric cancer tissues, and depicted that dysregulated circRNAs associated with gastric cancer. Moreover, some circRNAs can stably exist in human body fluid, and has the potential to be used as biomarkers for the screening of people with highrisk of gastric cancer.

## Ethical Approval

This study was approved by the Human Research Ethics Committee of Ningbo University School of Medicine (IRB No.20100303).

## Conflict of Interest

The authors made no disclosures.
